# Increase in Pneumococcus Macrolide Resistance, United States

**DOI:** 10.3201/eid1508.081187

**Published:** 2009-08

**Authors:** Stephen G. Jenkins, David J. Farrell

**Affiliations:** Weill Cornell Medical College, New York, NY, USA (S.G. Jenkins); Quotient Bioresearch Ltd, London, UK (D.J. Farrell)

**Keywords:** Streptococcus pneumoniae, streptococci, macrolides, surveillance, PROTEKT US, respiratory infections, bacteria, antimicrobial resistance, United States, research

## Abstract

During year 6 (2005–2006) of the Prospective Resistant Organism Tracking and Epidemiology for the Ketolide Telithromycin surveillance study, 6,747 *Streptococcus pneumoniae* isolates were collected at 119 centers. The susceptibility of these isolates to macrolides was compared with data from previous years. Macrolide resistance increased significantly in year 6 (35.3%) from the stable rate of ≈30% for the previous 3 years (p<0.0001). Macrolide resistance increased in all regions of the United States and for all patient age groups. Rates were highest in the south and for children 0–2 years of age. Lower-level efflux [*mef*(A)]–mediated macrolide resistance decreased in prevalence to ≈50%, and highly resistant [*erm*(B) + *mef*(A)] strains increased to 25%. Telithromycin and levofloxacin susceptibility rates were >99% and >98%, respectively, irrespective of genotype. Pneumococcal macrolide resistance in the United States showed its first significant increase since 2000. High-level macrolide resistance is also increasing.

## Abstract 2

During year 6 of the study, the incidence rate rose from ≈30% to 35.3%.

Antimicrobial drug treatment of community-acquired respiratory tract infections (RTIs) is usually initiated when the causative pathogen has not been documented. Treatment is therefore chosen empirically on the basis of potential pathogens and their antimicrobial susceptibility. *Streptococcus pneumoniae* is the major pathogen responsible for community-acquired RTIs (*1*), and treatment guidelines advise the use of agents that provide adequate coverage of this pathogen (*2*).

Although macrolides such as azithromycin and clarithromycin are active against *S. pneumoniae* and are in widespread clinical use, increasing in vitro bacterial resistance may have compromised their use. Resistance to macrolides in *S. pneumoniae* increased steadily during the 1990s; however, recent surveillance studies indicate that resistance may have plateaued at ≈30% in the United States (*3**–**5*). Although the link between in vitro resistance and clinical outcome is not fully understood, recent studies provide evidence that infection with macrolide-resistant pneumococci is a notable risk factor for failure of macrolide therapy in community-acquired RTIs (*6**–**9*).

Resistance to macrolides in *S. pneumoniae* is mediated by 2 major mechanisms: target modification caused by a ribosomal methylase encoded by the *erm*(B) gene or drug efflux encoded by the *mef*(A) gene. High-level macrolide resistance (MIC required to inhibit growth in 90% of organisms [MIC_90_] >32 μg/mL) is usually associated with *erm*(B), whereas *mef*(A)-mediated resistance, the most prevalent mechanism in the United States (*10*), usually results in lower-level resistance (MIC_90_ 1–4 μg/mL) (*11**,**12*). Results from the Prospective Resistant Organism Tracking and Epidemiology for the Ketolide Telithromycin (PROTEKT US) surveillance study, covering isolates collected during 2000–2004, indicate that the prevalence of *mef*(A) is decreasing, and isolates harboring *erm*(B) and *mef*(A) genes are becoming increasingly common (*13*). In addition, isolates carrying only the *mef*(A) gene showed a higher-level resistance (MIC_90_ = 16 μg/mL) than observed previously (*10*). This analysis reports results from year 6 of PROTEKT US (2005–2006), focusing on macrolide-resistance rates and mechanisms in *S. pneumoniae* isolates collected from patients with community-acquired RTIs.

## Methods

To reduce bias when interpreting trends, we restricted the analysis to *S. pneumoniae* isolates collected from the 119 centers that had previously provided isolates for year 5 of the study. Isolates were collected from patients in whom clinical acute/chronic bacterial sinusitis, acute/chronic otitis media, acute bacterial exacerbations of chronic bronchitis, chronic obstructive pulmonary disease, or community-acquired pneumonia had been diagnosed. Specimen sources included ear, blood, bronchoalveolar lavage, sinus aspirate, and sputum. Isolates were included from adults and children.

MICs for the antimicrobial agents were determined at the Central Microbiology Institute (CMI; Portland, OR, USA) by using the Clinical and Laboratory Standards Institute (CLSI) broth microdilution method (*14*) and were interpreted by using CLSI breakpoints (*15*). The breakpoints used for amoxicillin-clavulanate were <2 μg/mL (susceptible), 4 μg/mL (intermediate), and >8 μg/mL (resistant). Breakpoints used for cefpodoxime were <0.5 μg/mL (susceptible), 1 μg/mL (intermediate), and >2 μg/mL (resistant). Erythromycin-resistant (MIC >1 μg/mL) isolates were analyzed for the presence of *erm*(B), *mef*(A), and *erm*(TR) macrolide resistance genes by using a multiplex TaqMan PCR assay (*16*).

## Results

Of 6,747 *S. pneumoniae* isolates collected at 119 centers in year 6 of PROTEKT US, 2,381 (35.3%) showed in vitro resistance to erythromycin; this result compares with 1,907/6,257 (30.5%) in year 5. Resistance rates for azithromycin and clarithromycin in year 6 were 35.3% and 35.2%, respectively. Erythromycin resistance was stable at ≈30% in years 3, 4, and 5. Analysis of isolates from centers common to years 3–6 showed a significantly higher rate for year 6 than for years 3–5 (p<0.0001 by χ^2^ test).

Erythromycin resistance varied considerably by geography; the highest rates were in the North Central, Southeast, and South Central regions (Table 1). However, the rate of resistance increased from year 5 and year 6 in all 6 regions (Table 1).

**Table 1:** Erythromycin resistance among *Streptococcus pneumoniae* isolates, year 5 (2004–2005) and year 6 (2005–2006) of the PROTEKT US surveillance study*

US region†	Isolates, no. resistant/no. submitted (%)	
	Year 5	Year 6
Northeast	518/1,931 (26.8)	662/2,102 (31.5)
North Central	467/1,314 (35.5)	568/1,395 (40.7)
Northwest	94/417 (22.5)	108/422 (25.6)
Southeast	340/998 (34.1)	419/1,064 (39.4)
South Central	402/1,149 (35.0)	529/1,368 (38.7)
Southwest	86/448 (19.2)	95/396 (24.0)
Total	1,907/6,257 (30.5)	2,381/6,747 (35.3)

*PROTEKT US, Prospective Resistant Organism Tracking and Epidemiology for the Ketolide Telithromycin, United States.    †States submitting isolates for testing included Northeast: CT, DE, IN, MA, MD, MI, NJ, NY, OH, PA, RI, VT, and DC; North Central: IA, IL, KS, MN, MD, ND, NE, SD, and WI; Northwest: AK, ID, MT, OR, WA, and WY; Southeast: FL, GA, KY, NC, SC, VA, and WV; South Central: AL, AR, LA, OK, TN, and TX; Southwest: AZ, CA, CO, NM, NV, and UT.

**Figure 1:**
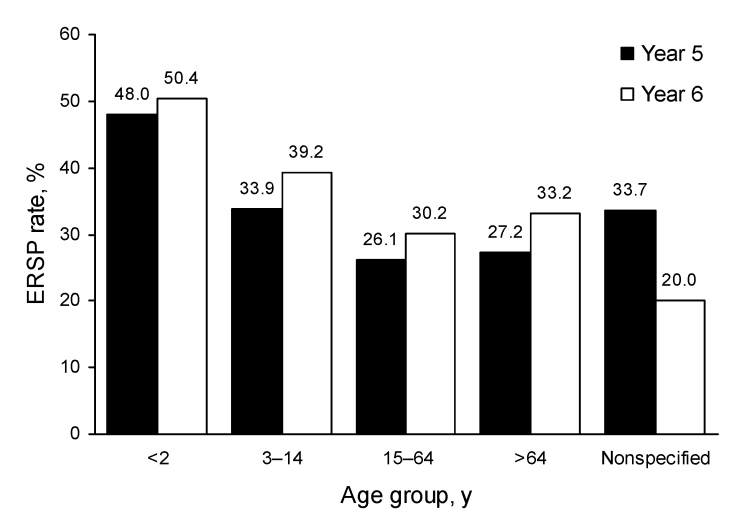
Increased prevalence of erythromycin-resistant *Streptococcus pneumoniae* (ERSP), by age group, Prospective Resistant Organism Tracking and Epidemiology for the Ketolide Telithromycin, United States surveillance study, years 1–6 (2000–2006).

Erythromycin resistance increased from year 5 to year 6 in all patient age groups; the highest rates of resistance occurred in isolates collected from children 0–2 years of age (year 5: 423/882 [48.0%]; year 6: 533/1,058 [50.4%]) (Figure 1).

Erythromycin resistance was less frequent in isolates collected from blood than in those collected from other sources (487/1,801 [27.0%] vs. 1,894/4,946 [38.3%]).The proportion of erythromycin-resistant *S. pneumoniae* (ERSP) isolates exhibiting high-level resistance to erythromycin (MIC >32 μg/mL) was 18.0% in year 6 compared with 13.4% in year 5.

Coresistance to penicillin (oral penicillin V for nonmeningitis isolates) was exhibited by 14.8% of ERSP isolates collected in year 6 compared with 13.2% of ERSP isolates collected at the same centers in year 5. Among all *S. pneumoniae* isolates, resistance to amoxicillin-clavulanate increased from 5.2% in year 5 to 8.1% in year 6; resistance to the third-generation oral cephalosporin, cefpodoxime, increased less substantially (19.4% in year 5 vs. 20.5% in year 6).

### Genotyping

The distribution of genotypes among ERSP isolates changed from year 5 to year 6. Lower-level efflux *mef*(A)-mediated macrolide resistance decreased, while high-level *erm*(B) with or without *mef*(A) increased (Table 2). In year 6, just over half of ERSP isolates showed *mef*(A) resistance; nearly one quarter were positive for *erm*(B) and *mef*(A). Analysis of isolates from centers common to years 3–6 of the study indicated a significant decreasing prevalence of *mef*(A) and significantly increasing prevalence of *erm*(B) ± *mef*(A) (p<0.0001 and p = 0.0033, respectively, by χ^2^ test).

**Table 2:** Macrolide resistance genotypes, year 5 (2004-2005) and year 6 (2005–2006) of the PROTEKT US surveillance study*

Genotype	No. isolates (% of ERSP)†	
	Year 5 (n = 1,907)	Year 6 (n = 2,381)
*erm*(B)	310 (16.3)	448 (18.8)
*mef*(A)	1,172 (61.5)	1,282 (53.8)
*erm*(B) + *mef*(A)	377 (19.8)	575 (24.1)
*erm*(TR)	2 (0.1)	0
Ribosomal mutations	26 (1.4)	41 (1.7)

*PROTEKT US, Prospective Resistant Organism Tracking and Epidemiology for the Ketolide Telithromycin, United States; ERSP, erythromycin-resistant *Streptococcus pneumoniae.*    †A total of 20 isolates in year 5 and 35 isolates in year 6 were not viable for genotyping.

**Figure 2:**
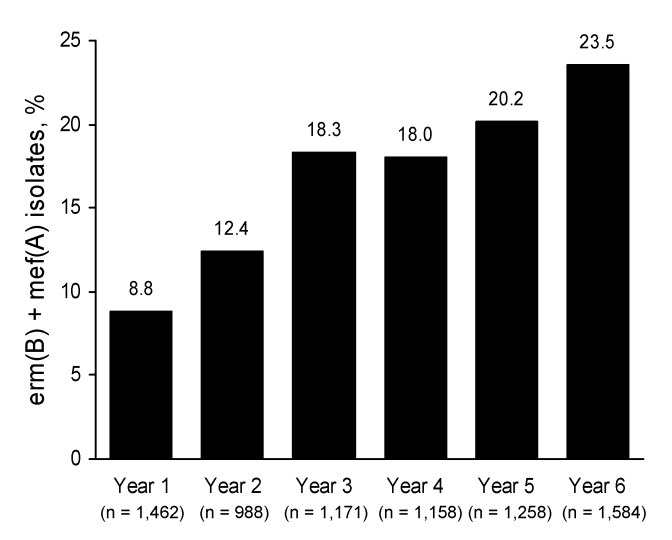
Increased prevalence in the *erm*(B) + *mef*(A) macrolide resistance genotype from year 1 (2000–2001) to year 6 (2005−2006), Prospective Resistant Organism Tracking and Epidemiology for the Ketolide Telithromycin, United States surveillance study.

ERSP isolates from patients 0–2 years of age showed the highest frequency of the *erm*(B) + *mef*(A) genotype (38.6% in year 6 compared with 35.5% in year 5). With the exception of years 3–4, the proportion of isolates harboring *erm*(B) and *mef*(A) throughout the 6 years of the PROTEKT US study has trended upward (Figure 2).

Most (398/575 [69.2%]) of the *erm*(B) + *mef*(A) isolates from year 6 were serotype 19A; most of the remainder (154/575 [26.8%]) were serotype 19F. Overall, 72.8% of year 6 ERSP isolates were susceptible to amoxicillin-clavulanate. However, amoxicillin-clavulanate susceptibility varied considerably between genotypes; <10% of isolates carrying *erm*(B) and *mef*(A) genes were susceptible to this agent compared with >90% of isolates harboring either gene alone (Table 3). MIC_50_ and MIC_90_ for amoxicillin-clavulanate among *erm*(B) + *mef*(A) ERSP isolates were both >8 μg/mL; these were also the values for 19A and 19F strains. By contrast, >98% of ERSP isolates were susceptible to levofloxacin and >99% were susceptible to telithromycin. The genotypic mechanism of erythromycin resistance had little impact on susceptibility to either of these agents (Table 3).

**Table 3:** Antimicrobial susceptibility of erythromycin-resistant isolates, by genotype, year 6 (2005–2006) of the PROTEKT US surveillance study*

Genotype	Amoxicillin−clavulanate							Levofloxacin							Telithromycin					
	Susceptibility, %				MIC, μg/mL			Susceptibility, %				MIC, μg/mL			Susceptibility, %				MIC, μg/mL	
	S	I	R		MIC_50_	MIC_90_		S	I	R		MIC_50_	MIC_90_		S	I	R		MIC_50_	MIC_90_
*erm*(B)†	96.2	0.9	2.9		0.12	2		98.2	0.2	1.6		1	1		99.1	0.7	0.2		0.03	0.12
*mef*(A)‡	92.1	3.7	4.2		0.25	2		98.9	0.1	1.0		1	1		99.6	0.4	0		0.25	0.5
*erm*(B) + *mef*(A)§	9.7	9.9	80.3		>8	>8		98.8	0.2	1.0		1	1		99.1	0.5	0.3		1	1

*PROTEKT US, Prospective Resistant Organism Tracking and Epidemiology for the Ketolide Telithromycin, United States; S, susceptible; I, intermediate; R, resistant.    †n = 448.    ‡n = 1,282.    §n = 575.

## Discussion

These findings from PROTEKT US indicate that pneumococcal macrolide resistance has demonstrated its first significant increase since the study began in 2000 (*4**,**13**,**17*). Whether the increase from ≈30% to 35% represents the start of a new upward trend will become evident only when results of surveillance studies in future years become available. However, it is worth noting that another smaller US surveillance study recently reported an azithromycin resistance rate of 34% in *S. pneumoniae* isolates collected during the same 2005–2006 respiratory infection season as this analysis (*18*). A further sustained rise in macrolide resistance would be a major cause for concern because macrolides, such as azithromycin and clarithromycin, remain in widespread use for the treatment of community-acquired RTIs in the United States.

The increase in ERSP isolates from year 5 to year 6 in all 6 regions of the country indicates a lack of specific local factors that might explain this sudden increase. Even so, resistance continued to be higher in some regions (Southeast, North Central, and South Central) than in others. Higher rates of macrolide resistance in the southern states aligns with a recent retrospective cohort study involving 1,574 patients with pneumococcal bacteremia, which identified residence in the southern United States as a risk factor for infection with macrolide-nonsusceptible pneumococci (*9*).

Other potential explanations for the increase in macrolide resistance include increased use and/or inappropriate prescription of macrolides. Pneumococcal macrolide resistance in *S. pneumoniae* has been linked in several studies with increased consumption of macrolides in general and of azithromycin in particular (*19*). Other factors associated with pneumococcal macrolide resistance are recent use of antimicrobial drugs, age extremes, and daycare attendance (*6**,**8*). However, which (if any) of these factors might explain the trends reported here are not clear.

Another concern arising from this report is the continuing change in the distribution of macrolide-resistance genotypes. Although *mef*(A), usually associated with lower-level macrolide resistance, remains the most prevalent genotype, it now accounts for only about half of all ERSP isolates. Isolates carrying *mef*(A) continue to be replaced by strains that harbor *mef*(A) and *erm*(B) genes. Data from the first 4 years of PROTEKT US showed that the proportion of *S. pneumoniae* isolates positive for *erm*(B) and *mef*(A) genes increased from 9.3% to 19.1% from Fall of 2000 through spring of 2001 and the same for subsequent years through spring of 2004 while isolates positive for the *mef*(A) gene decreased over this time from 69.0% to 60.7% (p = 0.03) (*10*). A Canadian study found a significant increase of 8% (from 4% to 12%) in the prevalence of dual *erm*(B) and *mef*(A)-positive *S. pneumoniae* isolates (p<0.05) between Fall and spring seasons of each year (1998–2004); this increase coincided with a 17% decrease in high-level *erm*(B)-mediated resistance and a 5% increase in the proportion of isolates carrying only the *mef*(A) gene (*20*). The latest PROTEKT US data show that these trends are continuing; nearly one quarter of year 6 ERSP isolates have both resistance genes, and the frequency of this genotype is approaching 40% in isolates from children.

The increased prevalence of isolates harboring *erm*(B) and *mef*(A) genes is most likely due to the recent expansion in the US and elsewhere of a multidrug-resistant serotype 19A pneumococcal clone that carries both resistance genes (*21**,**22*). The expansion of this clonal variant resistance provides at least a partial explanation for the greater frequency of high-level erythromycin resistance observed in year 6 compared with that of the previous year. Most *erm*(B) + *mef*(A) strains show high-level resistance to macrolides (MIC >32 μg/mL). In addition, although *mef*(A) is traditionally associated with lower-level macrolide resistance (MIC 1–4 μg/mL), recent data suggest that the macrolide MICs for a growing proportion of *mef*(A) isolates exceed 16 μg/mL (*10*).

Because most *erm*(B) + *mef*(A) strains show multidrug resistance (*21*), their increased prevalence may compromise the effectiveness of other commonly used antimicrobial therapies. For example, although >90% of all *S. pneumoniae* isolates collected in year 6 of PROTEKT US exhibited in vitro susceptibility to amoxicillin-clavulanate, <10% of *erm*(B) + *mef*(A) isolates tested in this analysis were susceptible to this agent. Moreover, the MIC distribution for amoxicillin-clavulanate within the *erm*(B) + *mef*(A) isolates (MIC_50_ and MIC_90_ both >8 μg/mL) suggests that no oral β-lactam antimicrobial drug may be available that can provide adequate concentrations to eradicate these increasingly prevalent strains. On the other hand, the fluoroquinolone levofloxacin and the ketolide telithromycin continue to show good activity against ERSP isolates, with little impact of resistance genotype on their respective activities.

Our study is subject to several potential limitations. A major potential limitation inherent in surveillance studies that measure in vitro antimicrobial drug resistance is their clinical relevance. Although an association between in vitro resistance and adverse clinical outcome remains generally unproven for most respiratory infections, an increasing number of studies indicate that infection with macrolide-resistant pneumococci is associated with clinical failure (*6**–**9**,**22*). Furthermore, clinical failures have been associated with *mef*(A)- and *erm*(B)-mediated resistance (*6**,**23**,**24*). A second potential limitation of this study is the derivation of resistance rates from collection centers where a predetermined number of isolates were to be collected and may not entirely reflect those found more widely.

These data from PROTEKT US year 6 indicate that in vitro pneumococcal macrolide resistance may not have plateaued as previously thought. Continued surveillance of erythromycin resistance in general, and of highly resistant *erm*(B) + *mef*(A) strains in particular, is warranted.
